# A Magnetic Soft Endoscopic Capsule-Inflated Intragastric Balloon for Weight Management

**DOI:** 10.1038/srep39486

**Published:** 2016-12-21

**Authors:** Thanh Nho Do, Khek Yu Ho, Soo Jay Phee

**Affiliations:** 1 California NanoSystems Institute (CNSI), University of California, Santa Barbara, Room 2810, Elings Hall, Mesa Road, CA, 93106, USA; 2 Department of Medicine, Yong Loo Lin School of Medicine, National University of Singapore and National University of Health System, 119260 Singapore; 3 School of Mechanical and Aerospace Engineering, Nanyang Technological University, 50 Nanyang Avenue, 639798 Singapore

## Abstract

Overweight and obesity have been identified as a cause of high risk diseases like diabetes and cancer. Although conventional Intragastric Balloons (IGBs) have become an efficient and less invasive method for overweight and obesity treatment, the use of conventional tools such as catheter or endoscope to insert and remove the IGBs from the patient’s body causes nausea, vomiting, discomfort, and even gastric mucous damage. To eliminate these drawbacks, we develop a novel magnetic soft capsule device with gas-filled balloon inflation. The balloon is made from a thin and biocompatible material that can be inflated to a desired volume using biocompatible effervescent chemicals. In addition, both the outer balloon and inner capsule are designed to be soft and chemical resistance. The soft capsule shell is fabricated using scaffold-solvent approach while the outer balloon utilizes a novel fabrication approach for 3D spherical structure. A prototype of the proposed capsule and balloon is given. Experiments are successfully carried out in stimulated gastric environment and fresh porcine stomach to validate the effectiveness and reliability of the proposed approach.

Overweight and obesity are a negative health issue that increase the chance of chronic degenerative diseases such as diabetes, gastrointestinal cancer or even death. More than 2 billion people worldwide had excess weight and more than 30% of them are obese according to the recent report from World Health Organization (WHO). To prevent the complicated diseases related to overweight and obesity, a decrease of 5% to 15% of the weight is highly recommended^1^. Various ways to promote the weight loss such as anti-obesity-drug, over-the-counter slimming herbs, and weight management program have been used^2^. Due to modernization and change in lifestyle patterns, a balance diet and regular exercises are much more challenging to maintain. For people with serious obesity, bariatric surgery is considered but this causes surgical complications such as kidney and metabolic diseases^3^. An alternative method is to use the Intragastric Balloon (IGB) to occupy the patient’s stomach and induce the feeling of satiety, then patient eats less and promote the weight loss^1^,^4^,^5^. To maximize efficacy and minimize hazardous issues during the treatment, the IGB should be removed every three months before continuing another IGB^6^. In some cases, IGB can be used up to one year^7^. It is recommended that the treatment period depends on the patient’s condition. For example, researchers indicated that around 55% of the patients continued losing at least 10% of their weight after 1 year-treatment^6^. Recently, 22-nonrandomized studies from 4371 patients demonstrated that the mean weight loss is around 17.8 kg after the first year of IGB treatment but around 20–40% of patients fail to lose weight during this period^8^. In similar research, a lost mean of 7.38 kg at the end of first month treatment and about 14.4 kg after six months of treatment was reported^5^. Dogon and his colleagues showed that the weight loss for obese patients after 6, 12, and 18 months were 50%, 71%, and 96%, respectively^9^. A significant weight-loss can be achieved for patients if they lose their weight greater than or equal 5% after 1 month. Normally, epigastric pain, discomfort, and nausea that develop in majority of patients occur a few hours after the IGB insertion. However, these side effects are relieved after few days^10^.

Although advantages, the use of conventional IGB requires endoscopic tools such as catheter or endoscope to place and remove the balloon from the stomach that cause patient’s discomfort, expensive, nausea, and vomiting^8^,^11^,^12^. In addition, a high cost for treatment is the main challenge. To overcome the complexity in the use of the conventional IGB, there are few developments for a non-invasive IGB in term of a pill or capsule. For example, Kencana *et al*.^13^. and Yan *et al*.^14^. developed a wireless capsule with on-board motor and electric circuit. Using remote magnetic control actuation, magnetically-driven micro devices, capsules, and drug delivery devices have been widely studied in the literature^15^,^16^,^17^,^18^,^19^,^20^,^21^,^22^,^23^. Do *et al*.^24^,^25^. also introduced a smaller capsule size using magnetic actuator with no limitations on the power supply. Although these proposed approaches did not require any endoscopic tools to remove and place the capsule in the stomach, many limitations still exist such as large size, higher risks for the patient, insufficient power supply, rigid capsule shell, complex inflation and deflation mechanism, and not biocompatible and chemical resistant for both balloon and capsule shell. To overcome these limitations, in this paper, we develop a new type of magnetic soft capsule endoscope that can control the inflation and deflation of a novel outer IGB. Three main features of the proposed soft capsule that outperform our previous approach include: (i) The whole capsule shells are soft, acid resistant, and biocompatible, (ii) the proposed magnetic soft capsule includes only an inner magnetic mechanism that can be used for both the inflation and deflation of the outer balloon compared to our previous approach where two inner magnetic mechanisms are required for both inflation and deflation phase, and (iii) the outer balloon which is obtained using novel fabrication method is acid resistant, soft, thin, biocompatible, and strong enough to resist the contraction force from the stomach motion.

Lighter weight of air-filled balloon has been proven by its effectiveness over the fluid-filled balloon due to its safer and longer occupation in the stomach^26^. Here we present a novel approach for the use of IGB in term of a magnetic soft capsule endoscope (MSCE) with the use of air-filled IGB to reduce the postoperative discomfort like epigastric pain, serve vomiting, and heaviness. To support the patient with more comfort and low cost, the whole capsule and its outer balloon are made from soft, biocompatible, and chemical resistant materials. Biocompatible effervescent chemicals are used to produce the carbon dioxide (CO_2_) gas and then inflate the outer balloon.

Soften and body compliances in medical applications are salient features to seek simplicity and reduce complexity in their working environment. Rigid body is often used extensively in a specific requirement to perform a single task efficiently^27^. During the robot and organ interaction, potential tissue damage because of the excessive magnetic force attraction from the rigid body could be a safety issue. All current capsules for obesity treatment are made of rigid outer surfaces. This creates very high stress and damage on the tissue. In addition, it can cause difficulties for liquid injection into the capsule shell. In contrast, soft structures have high deformation and light weight made out of intrinsically soft and biocompatible materials and can absorb much of energy arising from the collision. During the capsule-tissue interaction, soft structures make the capsule safer and less invasive. To design the magnetic soft capsule, its geometry and size is designed to be simple and as small as possible. The size of the inflated balloon should be larger than the polyric sphincter (normal human is around 13 ± 7 mm and 16 ± 8 mm for gastric ulcer patient^28^. In addition, the capsule should be able to hold a desired volume of acid for the reaction process. In this paper, we use a novel fabrication method for 3D PDMS capsule namely scaffold-solvent approach (SSA). In previous approaches for the capsule-inflated balloon, latex balloon was used for the validation. However, this type of balloon is not biocompatible and not chemical resistant. Therefore, it is not suitable for use in human gastric environment. Various fabrication techniques for making a monolithic and hollow structure such as injection^29^, rotational^30^, dip coating and spin coating^31^, and blow moulding^32^ are available. Because PDMS is relatively low elongation at break and this can cause difficulties to remove the silicone part from the 3D moulds, making a thin and flexible 3D balloon from PDMS is much more challenging for these methods. In addition, current fabrication techniques are limited in the shell thickness and uniformity. Although Lee *et al*.^33^. recently successfully fabricated a hemispherical structure using coating of curved surface method. However, a complete spherical structure is still lacked in this work due to the limitations on the removal method for the 3D mould. In addition, only PDMS layer is not strong enough to resist with the external force applied to the balloon. Here, we introduce a novel approach for the fabrication of a complete spherical PDMS balloon which is biocompatible and chemical resistant to the gastric environment. Especially, the new method allows a flexible control of the balloon size and thickness but simple, stable, and low cost.

To illustrate a potential clinical application for the proposed approach, we successfully carried out experiment on simulated gastric acid environment and also *ex-vivo* experiment on a fresh porcine stomach. The results demonstrated that the proposed soft capsule was able to inflate and deflated its outer balloon. In addition, its outer shells could resist well to the acid environment.

## Results

### Design Concept

**Figure 1:**
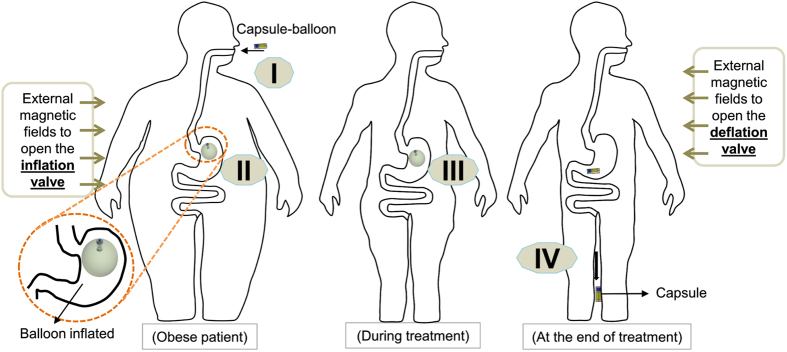
Schematic for the use of magnetic soft capsule.

In this paper, we use carbon dioxide (CO_2_) gas which is generated by a reaction between a biocompatible acid and base to inflate the outer balloon. The use of gas-filled balloon was proven to be safer and more efficient compared to fluid-filled balloon^10^. The detailed descriptions for selecting biocompatible acid and base as well as their optimal ratios were given in ref. 24. There are four main stages for the capsule usage as shown in Fig. 1. *Phase (I*)-patient swallows the soft capsule; *Phase (II*)-external magnetic field is introduced to open the inflation valve; *Phase (III*)-treatment period; and *Phase (IV*)-external magnetic field is introduced again to open the deflation valve and allows the capsule to naturally excrete out of the body.

### Soft capsule and balloon structures

**Figure 2:**
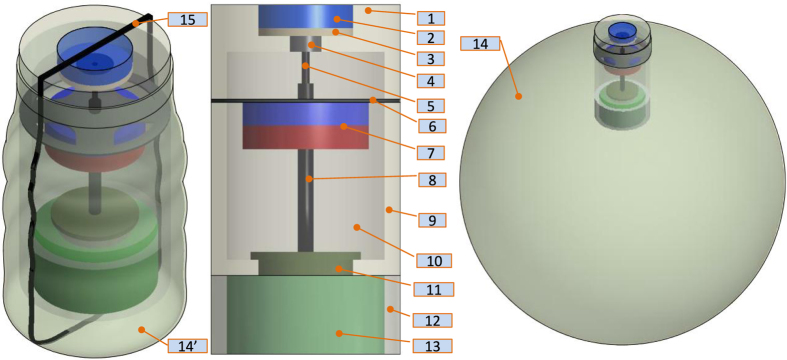
Magnetic soft capsule and balloon structures. (1) Inflation cover; (2) Nut for the deflation valve; (3) Chitosan layer; (4) Deflation valve; (5) Small fibre carbon rod; (6) Flexible membrane; (7) Internal magnet; (8) Big fibre carbon rod; (9) Acid chamber; (10) Acid; (11) Inflation valve; (12) Base chamber; (13) Base; (14) Inflated flexible balloon; (14’) Deflated flexible balloon; (15) flexible PDMS band.

In our previous approach^24^, the capsule was designed with two internal magnetic mechanisms for the inflation and deflation phases. In addition, the capsule shell is not chemical resistant in acidic environment and rigid. To reduce the capsule size and give more space for the chemical channels as well as simpler operation, we propose here a novel approach for the inflation and deflation using only an internal magnetic mechanism. The capsule shell is made by soft, biocompatible, and chemical resistant materials. This structure allows the patient to have more comforts and safer during the capsule-stomach interaction. The detailed magnetic soft capsule structure and its outer balloon are shown in Fig. 2. It consists of an inner soft capsule and an outer balloon. The acid chamber 9, base chamber 12, and magnetic inflation/deflation mechanism are located inside the soft capsule while the base is put outside the capsule but inside the balloon. To separate the two chemicals, an inflation valve 11 which is connected a small inner permanent magnet 7 via a hollow fibre carbon rod 8 is used. The magnet is directly sealed to a flexible PDMS membrane 6. This membrane allows the inner magnet to deform to its original position in the absence of external magnetic field. There is also a deflation valve 4 that allows the CO_2_ gas to exit when it is opened. At initial stage, the valve 4 is hold by a PDMS nut 2 and a thin layer of chitosan 3 via flexible PDMS band. A carbon fibre rod 5 is connected to the deflation valve. Specifically, the rod 5 is inserted in to the hollow rod 8. This insertion plays a role of guidance the inner magnet to slide along the axial direction of the capsule. The outer balloon is a type of flexible, soft, thin, and chemical resistant material. It is able to hold the CO_2_ gas and inflate to a desired volume.

### Inflation and deflation principle for the novel magnetic soft capsule-balloon

The CO_2_ gas which is generated by a reaction between citric acid 60% and potassium bicarbonate is used to inflate the outer balloon^24^. To create the chemical reaction, the inflation valve 11 must be opened. In this paper, we use an external permanent magnet to actuate the inner capsule. The use of permanent magnet has been proven to be safer and provides higher force compared to electromagnet^34^. The patient swallows the MSEC with a glass of water (*Phase I*). After a period of around 6 s, the capsule will reach the patient’s stomach via oesophageal peristalsis^35^. The total force F_I_ applied to the inflation mechanism is given in Eq. (1):


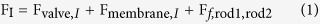


where F_valve,I_ is the force that requires to open only the inflation valve, F_membrane,I_ is the deformation force from the flexible membrane for inflation phase, and F_f,rod1,rod2_ is the friction force between the fibre rod 5 and 8.

**Figure 3:**
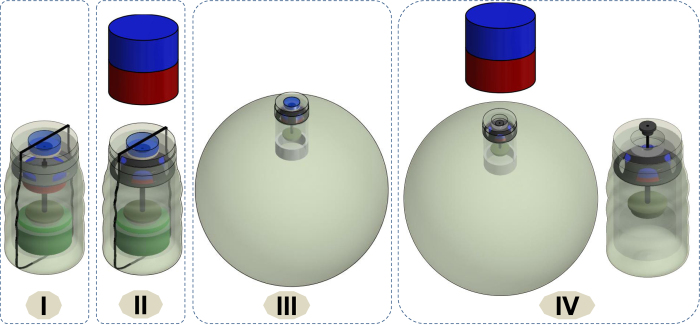
Inflation and deflation principle, (**I**) Capsule and outer balloon; (**II**) Inflation phase; (**III**) Treatment period; (**IV**) deflation phase.

The inflation and deflation principle is shown in Fig. 3. Once external magnet is gradually brought closer to the stomach, the capsule will move towards the external magnet (*Phase II*). Let FI_open_ is the force exerted to completely open the inflation valve. When F_I_ falls below FI_open_, this magnetic force will align the capsule itself in the same poles with the external magnet. This force also causes the capsule inflation tip to be braced against the inner lining of stomach wall. However, no inner motion is obtained during this stage. When F_I_ is greater than or equal FI_open_, the inner magnet and the flexible membrane start to move axially towards the external magnet. The inflation valve is opened and the acid from the liquid chamber is released to the base containing chamber. As a result, CO_2_ gas generated from the chemical reaction will thus inflate the outer balloon to a desired volume. The main purpose of flexible membrane 6 is to guide and hold the inner magnet to move axially and reform it to the original position. It is noted that the inner magnet is not able to open the deflation valve when the outer balloon is not fully inflated. The PDMS nut 2 and chitosan layer 3 plays a role of sealing and holding the valve 4. Initially, the nut 2 is hold by a flexible PDMS band 15. When the balloon is fully inflated, it will break this band and allows the nut 2 to fall off from the capsule leaving the valve 4 alone. The chitosan layer 3 will also be dissolved in the gastric acid environment after few hours.

The operation principle for the deflation process is similar to that of the inflation process. In brief, after treatment period raging from three to six months where the capsule balloon is in phase III, the external magnet gradually approaches the stomach and deflates the balloon by opening the deflation valve. At this stage, the nut 2 is separated from the capsule and the chitosan layer 3 is also dissolved in gastric environment. With no more support from the nut 2 and chitosan layer 3, the deflation valve will be opened by a threshold of deflation force FD_open._ The total forces F_D_ that apply to the deflation valve can be expressed by Eq. (2).


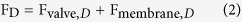


where F_valve,D_ is the force that requires to open only the deflation valve, F_membrane,D_ is the deformation force from the flexible membrane for deflation phase.

Note that the force given by Eq. (2) is the force where the inner magnet starts to push against the deflation valve. When F_D_ falls below FD_open_, this magnetic force will align the capsule itself in the same poles with the external magnet. This force also causes the capsule inflation tip to be braced against the inner lining of stomach wall. When F_D_ is greater than or equal to the force FD_open_, the inner magnet and the flexible membrane start to move axially towards the external magnet. The deflation valve is completely opened. As a result, the CO_2_ gas will exit the balloon and release to the stomach environment. The balloon and capsule reform to their original size and naturally excrete out of the patient body. Detailed calculations for the attraction forces will be given in the next sections.

### 3D printed scaffold-solvent approach for the soft capsule fabrication

**Figure 4:**
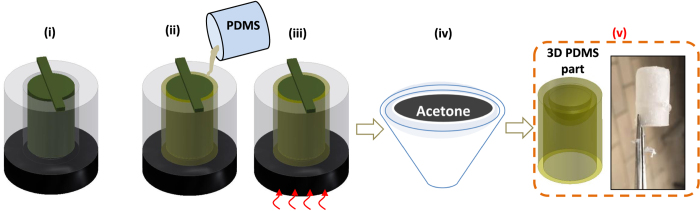
Scaffold-solvent method for 3D-PDMS fabrication. (**i**) Create 3D ABS mould from 3D printer; (**ii**) Pour PDMS to the mould; (**iii**) Heat to cure the PDMS; (**iv**) Dissolve the mould into Acetone; (**v**) Obtain the PDMS part.

Polydimethylsiloxane (PDMS) is one of the most commonly biocompatible and organic acid-resistant polymer materials in research laboratories. Its permeability is independent of the thickness if this threshold is above 50μm^36^. However, its limitation is relatively low elongation at break and this can cause difficulties to remove the silicone part from the 3D moulds. Achieving a 3D PDMS parts using standard fabrication methods is much more challenging. Here we use an off-the-shelf plastic polymer acrylonitrile butadiene styrene (ABS) to make the 3D moulds in which liquid PDMS is poured onto the 3D moulds to make to 3D capsule parts. The fabrication process for the 3D PDMS parts which is shown in Fig. 4 is divided into four main steps: making 3D mould, PDMS casting, curing, and PDMS-inner solvent. We choose Acetone for dissolving the ABS because the swelling ratio for PDMS in Acetone is around 1.06^37^. The final 3D PDMS parts from the SSA are put into distilled water and a pincer is used to remove the rest of undissolved ABS.

### Novel 3D fabrication method for the outer PDMS sphere balloon

A series of the coating process to form a sphere PDMS balloon is presented in Fig. S4, Fig. S5, and Fig. S6 (See Supplementary Information). A liquid Ecoflex 00–30 from Smooth-On Inc. which is obtained from mixing with a weight ratio 1:1 between the part A and part B is poured onto a sphere magnet (see Fig. S5(i) to Fig. S5(iii)). The obtained results are drained under the effect of gravity and eventually cured to form the Ecoflex sphere surface (see Fig. S5(iv)). Because the Ecoflex 00–30 has a high elongation of 900%, it is easy to peel off the elastic shell from the sphere surface (see Fig. S5(v) and Fig. S5(vi)). To obtain a desired volume PDMS sphere balloon, the Ecoflex shell is inflated by air. Liquid PDMS with a mixing ratio 10:1 for the elastomer and curing agent is poured onto the inflated Ecoflex shell, drained under the gravity, and subsequently cured in the oven at 75 °C for 4 hours (See Fig. S5(vii) to Fig. S5(x)). Finally, the balloon is obtained and ready for use with the proposed magnetic soft capsule. In order to enhance the strength of the balloon, sewing thread (SPOTLIGHT Pte Ltd, Singapore) with diameter of around 0.1 mm is used to wrap around the outer balloon. This additional work enhances the strength of the balloon (see Fig. S6 and Fig. S7 in Supplementary Information for more details). It is noted that the thickness 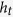 of the sphere balloon can be estimated from equations which are introduced in the Supplementary Information.

**Figure 5:**
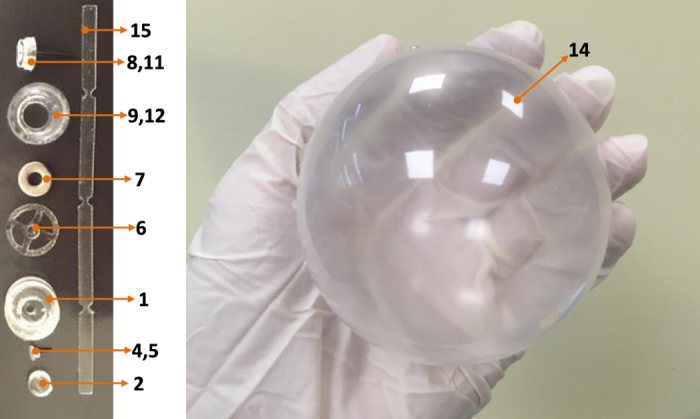
Fabrication results for inner soft capsule parts and outer balloon.

The novel fabrication method allows for flexible control of the 3D PDMS balloon thickness and its diameter at any sizes. To the best of our knowledge, this is the first time a simple fabrication method for the 3D PDMS sphere balloon with any sizes and thickness is introduced. Compared to dip coating method where a sphere mould is required, it is extremely challenging to remove the mould from the sphere PDMS shell. Therefore, our proposed fabrication method here offers a simpler and cheaper alternative. The obtained 3D capsule parts and PDMS sphere balloon are shown in Fig. 5.

### Characterization for the balloon inflation and deflation in a fresh porcine stomach

**Figure 6:**
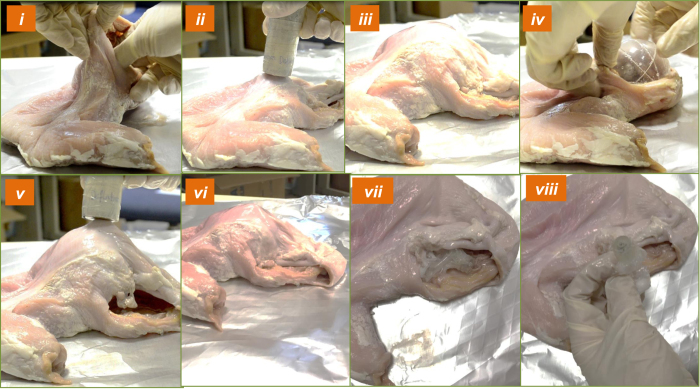
Validation on a fresh porcine stomach; (**i**) Insert the capsule-balloon into the porcine stomach; (**ii**) Introduce the external magnet to the porcine stomach; (**iii**) Chemical reaction and balloon is fully inflated; (**iv**) The inflated balloon is partially exposed from the porcine stomach for observation; (**v**) External magnet is introduced again for the balloon deflation; (**vi**) Deflation valve is opened and balloon is deflated; (**vii**) Balloon is completely deflated; (**viii**) Deflated balloon and its inner capsule are removed from the porcine stomach.

The capsule and balloon performances are also evaluated in a fresh porcine stomach obtained from Sheng Siong Supermarket Pte Ltd Singapore (7 Jurong West Avenue 5, Singapore 649486). The acid chamber is also filled with 1.1 ml of citric acid 60% and 0.45 ml of the base in the base chamber. The balloon capsule is put inside the stomach and external magnet gradually approaches the capsule. For this validation, the chitosan layer 3 is absented in this validation because there is no gastric acid available in the fresh porcine stomach. As shown in Fig. 6, the proposed capsule could open the inflation valve to create the chemical reaction and produce the CO_2_ gas for the balloon inflation. For the deflation, we lift up the capsule balloon to separate the nut 2 and the external magnet is introduced again. It could also see that the deflation can be opened easily when the external magnet is put closely to the capsule.

## Discussion

The idea of using an intragastric balloon to occupy the space in the stomach and give the feeling of satiety have been studied and successfully implemented in the past few years^1^,^38^,^39^,^40^,^41^,^42^. The presence of the IGB in the stomach delays gastric emptying, causing a premature sensation of satiety and thus results in decreased food consumption^10^. Conventional IGBs for obesity and overweight treatment have shown critical limitations such as high cost, discomfort, nausea, vomiting, and complex endoscopic procedures due to the requirement of insertion and removal tools^43^,^44^,^45^. To maximize the efficacy for treatment and to minimize the side effects, the IGB should be removed every three months^6^. Depend on the patient’s condition, the treatment period ranges from three months to one year^7^. Normally, epigastric pain, discomfort, and nausea that develop in a majority of patients occur a few hours after balloon insertion. However, these effects will be relieved after few days^10^. The removal and insertion procedures for the new IGB associate with very high cost and complications due to the need of complex tools and endoscopic team.

In this paper, we developed a magnetic soft capsule robot for overweight and obesity treatment. The proposed approach is a procedureless method without requiring any complex endoscopic tools and endoscopists for the balloon placement and removal after the treatment. In addition, the use of air-filled balloon for inflation approach offers many benefits over the fluid-filled balloon such as high safety, less epigastric pain, and longer occupation time, and light weight^26^,^39^. Previous approaches on the capsule endoscopy for obesity treatments were also limited in the rigid capsule shells that cause damage and pain to the stomach one during the capsule-tissue interaction. In addition, the capsule material is not biocompatible and acid resistant. We here developed a world’s first magnetic soft capsule robot for obesity and overweight treatment. The capsule shell and its inner complex soft structures are biocompatible and acid resistant and were fabricated using a novel fabrication method namely scaffold-solvent approach. Instead of using other conventional method like rotating, injection, or spin-coating, we utilized the solvent property of commercial polymer (ABS) in Acetone to obtain the complex soft capsule structures. Although we can achieve soft structures for the PDMS capsule using conventional technique like injection moulding, however, we have to align and bond them together using special equipment and tools. These works require additional steps and times. Compared to this, solvent casting method is quite efficient since the ABS materials from 3D printer and Acetone are quite cheap. Therefore, we can freely fabricate any complex inner shapes. In addition, we can mass produce these parts without concerning to the align and inner complex structures. While current IGB is limited in large thickness and high cost, we also developed a novel intragastric balloon for use together with the soft capsule. We used a novel fabrication method namely coating layer surface of a sphere structure to obtain a biocompatible and acid resistant sphere balloon. This is the first time a novel PDMS sphere balloon is introduced for any size and thickness without requiring the complex fabrication methods like rotating or dip coating. The balloon fabrication approach can open potential benefits to the IGB approach with an easy way and lower cost for clinical applications. While current gas-filled IGBs used a tube/catheter for the balloon inflation, we use on-board effervescent biocompatible chemicals and magnetic actuator to produce the carbon dioxide and inflate the balloon. This offers a wireless inflation for the balloon without requiring any connector between the balloon and the external environment. In addition, the limitations on the power supply for the capsule are completely eliminated since we use permanent magnetic fields for the actuation. As a result, the capsule can stay inside the patient’s body for a very long time.

For safety, a sensor network is needed to determine the capsule position after it is swallowed. It has been reported in the literature that a magnetic sensor network can be easily obtained using hall effect sensors^46^. The capsule should be monitored from six to eight hours after it is swallowed. The magnetic sensor network will help to confirm whether the capsule has reached the stomach or not before an external magnetic field is introduced to inflate the balloon. Although we carried out experiments to demonstrate the mechanical strength of the balloon, a methylene blue powder should be placed inside the balloon to determine the physical states of the balloon. If the inflated balloon is suddenly broken, the blue power will be dissolved in the stomach environment and therefore the patient urine will be changed in colour. Actually, if the balloon is suddenly broken, it can easily pass through the polyric sphincter and naturally excrete out of the patient’s body. Our proposed capsule will intend to stay in the patient stomach for few days and patients can swallow other new capsules after the first capsule. Compared to current endoscopic method for the use of IGB, our capsule could be cheaper and easier in use. As a result, patients can swallow multiple capsules with more comfort.

It also reported that there are different gastric capacities for the human stomach. Kim *et al*.^47^. showed that there were no differences in gastric volumes, ratio of postprandial/fasting gastric volume, and maximum ingested volume between obese men and control men, or between obese women and control women. Geliebter *et al*.^48^,^49^,^50^. carried out experiments in different volunteer groups and concluded that the gastric capacity for a normal human is around 1017 ± 154 ml while that for obese patient is around 1920 ± 136 ml. For bariatric surgery, adjustable gastric band which is a vertical separation of the proximal stomach with a stapler forming a small chamber in the region of the cardia with a capacity of around 20 ml is preferred because it is helps to slow down the consumption of food and induce the weight-loss. For other bariatric surgery, depend on the patient’s condition, the stomach size can be reduced from 100 ml to 600 ml^51^,^52^. For intragastric balloon treatment, the stomach space is normally occupied by one or two balloons ranging from 150 ml to 600 ml or up to 1000 ml^4^. For our proposed capsule-balloons, the stomach space should be occupied by a volume ranging from 150 ml to 300 ml. Table S3 gives an overview on the human stomach size.

To conclude, we have demonstrated magnetic soft capsule robot capable of inflation to occupy the patient stomach for overweight and obesity treatment. The proposed soft capsule balloon offers a safer and effective method for obesity treatment as compared to other weight-loss methods without using complex endoscopic procedures and tools. To realize the soft, biocompatible, and chemical resistant for the capsule, we utilized the novel scaffold-solvent approach for making its complex inner structure. Compared to other conventional fabrication methods, this approach is easily carried out with a high reliability. In addition, to overcome the existing limitations on the fabrication of a biocompatible and chemical-resistant sphere balloon, we developed a novel fabrication methods based on coating curved surface method on an outer 3D sphere structure. The proposed method for the 3D sphere PDMS balloon fabrication offers flexible control the balloon shell thickness and diameters with any size. To the best of our knowledge, this is the first time a PDMS sphere balloon for obesity and overweight treatment is obtained using the proposed method. We successfully validated the capsule and balloon inflation and deflation on acidic environment and fresh porcine stomach. In the next phases of this research, we plan to carry out experiments on both living animals (pigs) and human with institutional review board applications once the efficacy and safety have been proven. Although we limited our validation and analysis for the inflation balloon with real-time measurement and experiment, FEM analysis for the inflation balloon should be carried out to determine optimal parameters for the balloon thickness and outer size.

## Methods

### Soft capsule and balloon fabrication

We used 3D printer Fotus 250mc from Stratasys Ltd. to make the 3D-ABS moulds for the inner capsule structure 1, 9, and 12. The liquid PDMS (SYLGARD ^®^ 184 Silicone Elastomer kit, Down Corning, USA) with a weight ratio 10:1 for the elastomer and curing agent after degassed to remove the air bubbles is poured onto the 3D moulds. Later, they are cured at 75 °C for 4 hours in a drying chamber (Model VD 23, BINDER, Germany). The obtained curing products are immersed in Acetone (Sigma Aldrich, USA) for 12 hours to dissolve the scaffold (ABS moulds). To fabricate the other parts such as nut 12, deflation valve 4, flexible membrane 6, inflation valve 11, and flexible PDMS band 15, 3D moulds which obtained from 3D-printer SLM^®^500HL from SLM Solution group AG, Germany. The liquid PDMS (SYLGARD ^®^ 184 Silicone Elastomer kit, Down Corning, USA) with a weight ratio 10:1 for the elastomer and curing agent after degassed to remove the air bubbles is poured onto the 3D moulds. Subsequently, they are cured at 75 °C for 6 hours in the drying chamber of the oven VD23 from BINDER. The obtained cured silicone parts are removed from the moulds and ready for use. For the balloon fabrication, both Ecoflex and PDMS are degassed 3 minutes at 25 °C in the vacuum before pouring onto the sphere surfaces. The sphere magnet which obtained from Giosis Pte Ltd, Singapore has a diameter of 25 mm. To make the second layer for the sphere PDMS balloon, the balloon with first layer is inverted inside-out and inflated to the desired volume. Then the steps from Fig. S5(vii) to Fig. S5(x) are repeated.

### Magnetic soft capsule and balloon assembly

An uncured liquid PDMS is used to connect the flexible membrane part 6 into a permanent ring magnet (NdFeB/N52). They subsequently are heated up to 75 °C for 6 hours. The hollow carbon fibre rod 8 is sealed with the inflation valve 11 by an uncured liquid PDMS. The obtained parts are connected to the ring magnet and the flexible membrane using liquid PDMS. Similarly, smaller carbon fibre rod 5 which is inserted into the inner hole of carbon rod 8 is also sealed to the deflation valve 4 using liquid PDMS. Once the connected parts are ready, the assembly process can be carry out in the following steps. Firstly, the combined parts 11, 8, 7, and 6 are inserted into the acid chamber where the valve 11 is fit on the hole of the acid chamber and the lower surface of flexible membrane 6 is sealed onto its upper surface using an uncured liquid PDMS. Secondly, the upper surface is also connected to the part 1 using uncured liquid PDMS. The whole capsule then is heated up to 75 °C for 6 hours to cure the connectors. Thirdly, the inflation valve 4 with its rod 5 is inserted into the capsule chamber where the rod 5 is put inside the hole of rod 8. Next, a thin layer of aqueous solution of chitosan (Sigma Aldrich, USA) is poured onto the upper side of the deflation valve where the nut 2 is inserted into the chitosan layer thereafter. Once the capsule parts are completely assembled, the potassium bicarbonate is put into the base chamber. The complete capsule is inserted into the new balloon and sealed with the uncured liquid PDMS, subsequently heated at 50 °C overnight. Then the citric acid is filled into the acid chamber using a syringe-needle and a needle (See Supplementary Information). Finally, the PDMS band 15 is wrapped around the capsule balloon to hold the nut 2.

### Force measurement setup

For characterization of the balloon strength, we use a weight scale machine from KERN & SOHN GmbH, UK. For both experiments, the slider is travelled for distance of 15 mm to create the compression force on the balloon (See Fig. S7 in Supplementary Information). For the inflation and deflation force measurement, we use a FUTEK loadcell LSB200 connected to NI cRIO-9076 and LABVIEW software to decode the recorded signals (See Fig. S9 in Supplementary Information). The 3 mm thickness internal magnet (Grade N52, NdFeB) is a ring-type magnet with an inner diameter and outer diameter of 3 mm and 8 mm, respectively. To provide the attraction force for the system, another permanent cylinder magnet with a diameter of 15 mm and thickness of 6 mm is used. We carried out five trials for each experiment and the obtained results are mean value from the five trials.

## Additional Information

**How to cite this article:** Do, T. N. *et al*. A Magnetic Soft Endoscopic Capsule-Inflated Intragastric Balloon for Weight Management. *Sci. Rep.* **6**, 39486; doi: 10.1038/srep39486 (2016).

**Publisher's note:** Springer Nature remains neutral with regard to jurisdictional claims in published maps and institutional affiliations.

## Supplementary Material

Supplementary Information
