# Supplementary material for: A Magnetic Soft Endoscopic Capsule-Inflated Intragastric Balloon for Weight Management
Source: Sci Rep. 2016 Dec 21;6:39486. doi: 10.1038/srep39486 (PMC5175216; doi:10.1038/srep39486)
Supplement: Supplementary Information [file srep39486-s1.pdf]

Supplementary Information

A Magnetic Soft Endoscopic Capsule-Inflated Intragastric Balloon for Weight Management

Thanh Nho Do[1,*], Khek Yu Ho[2], and Soo Jay Phee[3]

[1]California NanoSystems Institute (CNSI), University of California, Santa Barbara, Room 2810, Elings Hall, Mesa Road, CA, USA, 93106

[2]Department of Medicine, Yong Loo Lin School of Medicine, National University of Singapore and National University of Health System, Singapore 119260

[3]School of Mechanical and Aerospace Engineering, Nanyang Technological University, 50 Nanyang Avenue, Singapore 639798

[*] *Corresponding author*

Email: dothanhnho@engineering.ucsb.edu

1. Size estimation for flexible membrane

The flexible PDMS membrane 6 plays an important role for holding and guiding the inner magnet to slide axially the capsule shell. If the membrane is too large, it can cause difficulties for the sliding of inner magnet. In contrast, if the membrane is too small, it is not able to hold the magnet. Therefore, it is necessary to estimate its thickness *h* and width *b* in order to provide a rough dimension for the membrane fabrication. Here we apply a pseudo rigid body model for large deformation beam bending under fixed-clamped boundary condition to estimate the flexible membrane size [1]. We choose a symmetric profile for the membrane as shown in Fig. S1. The relation between the applied force $F_{applied}$ and its axial deflection x_ε can be described by:

$$F_{applied}=\frac{K_A \Delta L x_\varepsilon}{\Delta L+\gamma L_0}+\frac{2K_T\gamma L_0\tan^{-1}(x_\varepsilon/\gamma L_0)}{(\Delta L+\gamma L_0)^2} \quad \text{(S1)}$$

$$K_T = 2\gamma K_\theta (EI)_{bending}/L_0 \quad \text{(S2)}$$

$$K_A = (EA)_{axial}/(\Delta L+\gamma L_0) \quad \text{(S3)}$$

$$\Delta L = \sqrt{x_\varepsilon^2+(\gamma L_0)^2}-\gamma L_0 \quad \text{(S4)}$$

where K_A, K_T are the required stiffness of the modelled torsional spring and nonlinear axial spring, respectively. ΔL is the increase of the sliding length. K_θ is the stiffness coefficient. γ is the characteristic radius factor. L_0 is the length of original beam. $(EI)_{bending}$ is the flexural rigidity. $(EA)_{axial}$ is the force per unit strength. *E* is the elastic module of the material, $I = bh^3/12$ is the moment of inertia, $A = bh$ is the cross section area of the beam. *b* and *h* is the width and thickness of the membrane, respectively.

If there is no external force applied to the membrane, the membrane should be able to hold the inner magnet. In contrast, if a determined external force is introduced, the inner magnet should be able to slide for a desired distance and open the deflation valve. However, the total force applied to the capsule should be less than a threshold. If the required force is too big, the size of external

magnet will be very large [2]. In this paper, we choose this threshold is F_{thres}=0.6N. The maximum inflation force and deflation force to open the inflation valve and deflation valve are around 0.2N and 0.11N, respectively (see next sections for force calculation of inflation and deflation valve). In order to provide a lower modulus, a lower temperature for curing time of PDMS is chosen (here at 25^0C). At this curing temperature, the elastic Young' modulus E for PDMS is 1.32MPa [3]. Since the flexible membrane consists of 4 single beams (beams 1, 2, 3, and 4 as shown in Fig. S2), the force applied in the continuum model for a single beam $F_{applied} = F/4$ should be less than 0.4N which is equivalent to (F_{thres}-0.2N).

An experimental setup for the flexible membrane calculation is shown in the Fig. S2. We use 3D printer Fotus 250mc from Stratasys Ltd. to make the 3D mould. The liquid PDMS with a mixing weight ratio 10:1 between the polymer and curing agent is degassed in a vacuum chamber and poured onto the 3D mould. Subsequently, it is cured at 25^0C for 48 hours and the membrane is removed from the mould. As discussed in [3], the Young modulus for the PDMS membrane at curing temperature of 25^0C is 1.32Mpa. The dimensions for the membrane are chosen as follow: $b = 1.1mm, h = 0.17mm, L_0 = 1mm$. The parameter γ is chosen to be 0.95. The small membrane displacement x_ε is constrained into 1mm. The flexible membrane is fixed on a support and an external magnet is gradually approached to the inner magnet until it moves a distance of 1mm. At this distance, the weight machine shows 25g. It means that the force applied to the membrane at elongation of 1mm is F=25*9.81*0.001=0.24525N.

To find the value of K_θ for the PDMS membrane, we need to find K_T. It should be noted that the value for force calculation in Eq. S1 should divide by 4 (for 4 beams). From Eq. S1 to Eq. S4, the value of K_θ is 6.1337. Using this value, we can proceed to estimate the membrane thickness/width for the capsule.

With given conditions such as $K_\theta = 6.1337, \gamma = 0.95, L_0 = 1mm, \delta = 2mm$, we have:

$$6.8075\times10^5\times b\times h+5.601\times10^{11}\times b\times h^3\leq 0.1 \quad \text{(S5)}$$

The inequality from Eq. (S5) will be used for the fabrication process of membrane dimension. For example, if we choose $h=0.2mm$, then b should be less than 0.711mm.

2. Thickness estimation for the outer balloon

Let μ_0 is the viscosity of the coating polymer, ρ is the polymer density, g is the earth gravity, $\emptyset$ is the Zenith angle, and R is the radius of the sphere ball. The squared root dependence of the outer layer PDMS thickness for sphere balloon $h_{t,PDMS}$ on its radius R can be expressed as follow [4]:

$$h_{t,PDMS}=\sqrt{\frac{3\mu_0 R}{4\rho g K}}\,(1+0.1\emptyset^2) \quad \text{(S6)}$$

where $K=\frac{k-e^{-\beta\tau_c}}{\beta}+\frac{\tau_c e^{-\beta\tau_c}}{\alpha-1}$ with $k=e^{-\beta\tau_w}$ with the waiting time τ_w procedure between the preparation and the coating of the polymer or $k=1$ if there is no waiting time. τ_c is the curing time of the polymer, β and α are the fitting coefficient of viscosity curve.

As shown in Fig. S3, if we consider Ecoflex balloon with its thickness d_0 and the initial inner and outer radius of r_0 and R_0, respectively. Suppose that the Ecoflex material is incompressible. Then its thickness d_1 as well as inner radius r_1, outer radius R_1 after the Ecoflex balloon is inflated in relation with its initial dimensions can be expressed by [5]:

$$d_0 r_0^2=d_1 r_1^2 \quad \text{(S7)}$$

Or $d_1=d_0[\frac{r_0}{R_1-d_1}]^2$

where $d_1=R_1-r_1$ and $d_0=R_0-r_0$

Finally, the thickness d_1 of the inflated Ecoflex balloon can be found from the solution of the following equation:

$$d_1^3-2R_1 d_1^2+R_1^2 d_1-d_0 r_0^2=0 \quad \text{(S8)}$$

From Eq. (S7) and Eq. (S8), the final thickness h_t for both outer PDMS and inner Ecoflex balloon can be given as:

$$h_t = h_{t,PDMS} + d_1 \qquad \text{(S9)}$$

It is noted that the values of r_0 is know from the initial sphere ball. For the value d_0, it is easily obtained using the Eq. (S6) for Ecoflex material. The radius R_1 can be measured when the Ecoflex balloon is inflated to this size. The value d_0 can be derived from Eq. (S6) as follow:

$$d_0 = h_{t,Ecoflex} = \sqrt{\frac{3\mu_{0,Eco} r_0}{4\rho_{Eco} g K_{Eco}}} \left(1 + 0.1\emptyset_{Eco}^2\right) \qquad \text{(S10)}$$

where $K_{Eco} = \frac{k_{Eco} - e^{-\beta_{Eco}\tau_{c,\mathrm{Eco}}}}{\beta_{\mathrm{Eco}}} + \frac{\tau_{c,\mathrm{Eco}} e^{-\beta_{Eco}\tau_{c,\mathrm{Eco}}}}{\alpha_{Eco} - 1}$ with $k_{Eco} = e^{-\beta_{Eco}\tau_{w,Eco}}$ with the waiting time $\tau_{w,Eco}$ procedure between the preparation and the coating of the polymer or $k_{Eco} = 1$ if there is no waiting time. $\tau_{c,\mathrm{Eco}}$ is the curing time of the polymer, β_{Eco} and α_{Eco} are the fitting coefficient of viscosity curve. $\emptyset_{Eco}$ is the Zenith angle for the Ecoflex material case.

3. Characterization of external force applied to the outer balloon

The proposed outer balloon is inflated and subsequently tested in a force testing system. It was known that the force per contraction averaged is around 0.2N to 0.65N for the stomach applied to the food or hard capsule [6]. We applied a constant force of around 2.2N to the balloon and put them in solution of simulated gastric acid (pH=1.1-1.3, Sigma Aldrich, USA) for one day. The validation is also carried out for sewing thread balloon using an average force of around 4.2N. As shown in Fig. S7, the both balloons are able to resist with the applied force and even can resist with a bigger force than the contraction force from the stomach.

4. Characterization of the inflation and deflation force

We connect the capsule with its inflation and deflation valves to a force measurement system to provide the force information. Because the tip of carbon fibre rod 8 is located 2mm away from the

deflation valve, the PDMS membrane 6 has a maximum travel length of 2mm if the deflation valve is not opened. The inflation force is only recorded whenever the inflation valve is opened. This means that only the maximum force to open the inflation valve is considered. For the case of deflation force, we remove the nut 2 to allow the deflation valve can be moved towards the external magnet. Similar measurement is carried out for the deflation phase where only maximum force is recorded whenever the deflation valve is opened. We carried out five trials for each measurement and the results are shown in Table S1. It can be seen that the maximum force for the inflation and deflation (max) is around 0.5664N. To generate this force, a cylinder permanent magnet with the size of at least 300mm in length and 250mm in diameter is required. This estimated size is obtained using COMSOL Multiphysics 5.0 software for a distance of 50mm between the external cylinder magnet and the inner ring magnet. Both external and inner magnets have the strongest grade of neodymium N52. For a longer distance between the two magnets, see [2] and Figs. S9, S10 for more details.

5. Soft capsule and balloon in acidic environment

We characterize the chemical resistant of the capsule shell and outer balloon in acidic environment. The acid chamber is filled with 1.1ml of 60% of citric acid (see Fig. S11 for the detailed injection of the acid into the capsule chamber) and 0.45ml of potassium bicarbonate is placed inside the balloon but outside the acid chamber. We subsequently put the balloon and the capsule into a solution of simulated gastric acid (pH=1.1-1.3, Sigma Aldrich, USA) which is contained in a petri dish. An external cylinder permanent magnet gradually approaches the capsule balloon to open the inflation valve. The two chemicals are mixed each other to create the CO_2 gas and subsequently inflate the outer balloon. The PDMS band is broken down due to the increase of the balloon volume. It can be observed that the chitosan layer is completely dissolved in gastric acid and the nut 2 is also separated from the capsule under the absence of PDMS band. There are no leakages of acid or erosion of the capsule shell/ balloon. The outer diameter of the inflated balloon is around 70mm

which is larger than the normal diameter of the polyric sphincter. Hence the balloon is guaranteed to stay inside the stomach during the treatment. After a predetermined time (here around two hours), we introduce the external magnet to the capsule again. The deflation valve is opened and the CO_2 gas is released from the balloon. This confirms that the capsule-balloon are successfully inflated and deflated at desired times. The experiments for the balloon inflation and deflation in gastric acid are shown in Fig. S8.

References

1 Howell, L. L., DiBiasio, C. M., Cullinan, M. A., Panas, R. M. & Culpepper, M. L. A Pseudo-Rigid-Body Model for Large Deflections of Fixed-Clamped Carbon Nanotubes. *Journal of Mechanisms and Robotics* **2**, 034501-034501, doi:10.1115/1.4001726 (2010).

2 Do, T. N., Seah, T. E. T., Yu, H. K. & Phee, S. J. Development and Testing of a Magnetically Actuated Capsule Endoscopy for Obesity Treatment. *PLoS ONE* **11**, e0148035, doi:10.1371/journal.pone.0148035 (2016).

3 Johnston, I. D., McCluskey, D. K., Tan, C. K. L. & Tracey, M. C. Mechanical characterization of bulk Sylgard 184 for microfluidics and microengineering. *Journal of Micromechanics and Microengineering* **24**, 035017 (2014).

4 Lee, A. *et al.* Fabrication of slender elastic shells by the coating of curved surfaces. *Nat Commun* **7**, doi:10.1038/ncomms11155 (2016).

5 Müller, I. & Strehlow, P. *Rubber and Rubber Balloons: Paradigms of Thermodynamics*. (Springer Berlin Heidelberg, 2004).

6 Kong, F. & Singh, R. P. Disintegration of Solid Foods in Human Stomach. *Journal of Food Science* **73**, R67-R80, doi:10.1111/j.1750-3841.2008.00766.x (2008).

7 Geliebter, A. *et al.* Gastric capacity, gastric emptying, and test-meal intake in normal and bulimic women. *The American Journal of Clinical Nutrition* **56**, 656-661 (1992).

8 Geliebter, A. Gastric distension and gastric capacity in relation to food intake in humans. *Physiology & Behavior* **44**, 665-668, doi:http://dx.doi.org/10.1016/0031-9384(88)90333-2 (1988).

9 Geliebter, A. & Hashim, S. A. Gastric capacity in normal, obese, and bulimic women. *Physiology & Behavior* **74**, 743-746, doi:http://dx.doi.org/10.1016/S0031-9384(01)00619-9 (2001).

10 Farrell, T. M. *et al.* Clinical application of laparoscopic bariatric surgery: an evidence-based review. *Surgical Endoscopy* **23**, 930-949, doi:10.1007/s00464-008-0217-1 (2009).

11 Naik, R. D., Choksi, Y. A. & Vaezi, M. F. Consequences of bariatric surgery on oesophageal function in health and disease. *Nat Rev Gastroenterol Hepatol* **13**, 111-119, doi:10.1038/nrgastro.2015.202

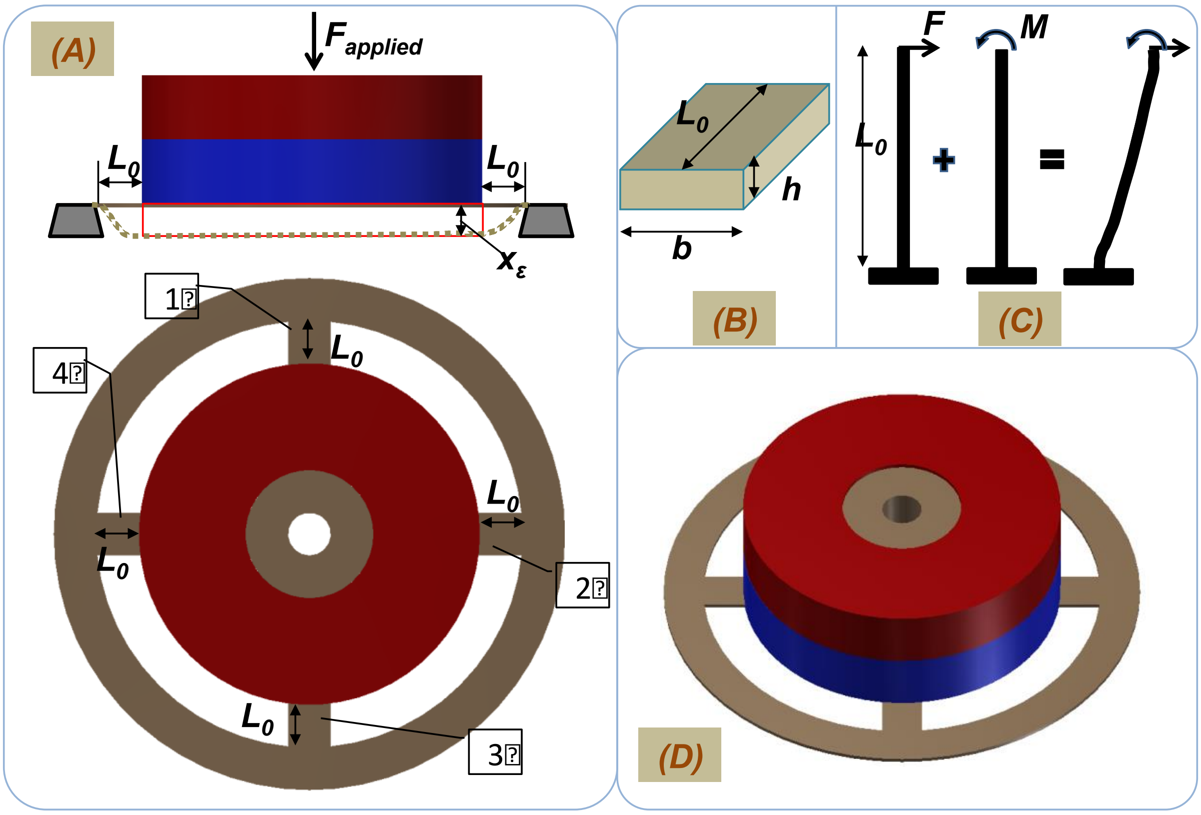

Fig.S1: Fixed-guide model for the flexible membrane with the inner magnet, (A) 2D view for the applied force and its deflection with four thin bar connected; (B) Cross section for the thin bar membrane; (C) Fixed-guided boundary condition with large axial stress induced by a bending force *F* and moment *M*; (D) 3D view of the flexible membrane and inner magnet.

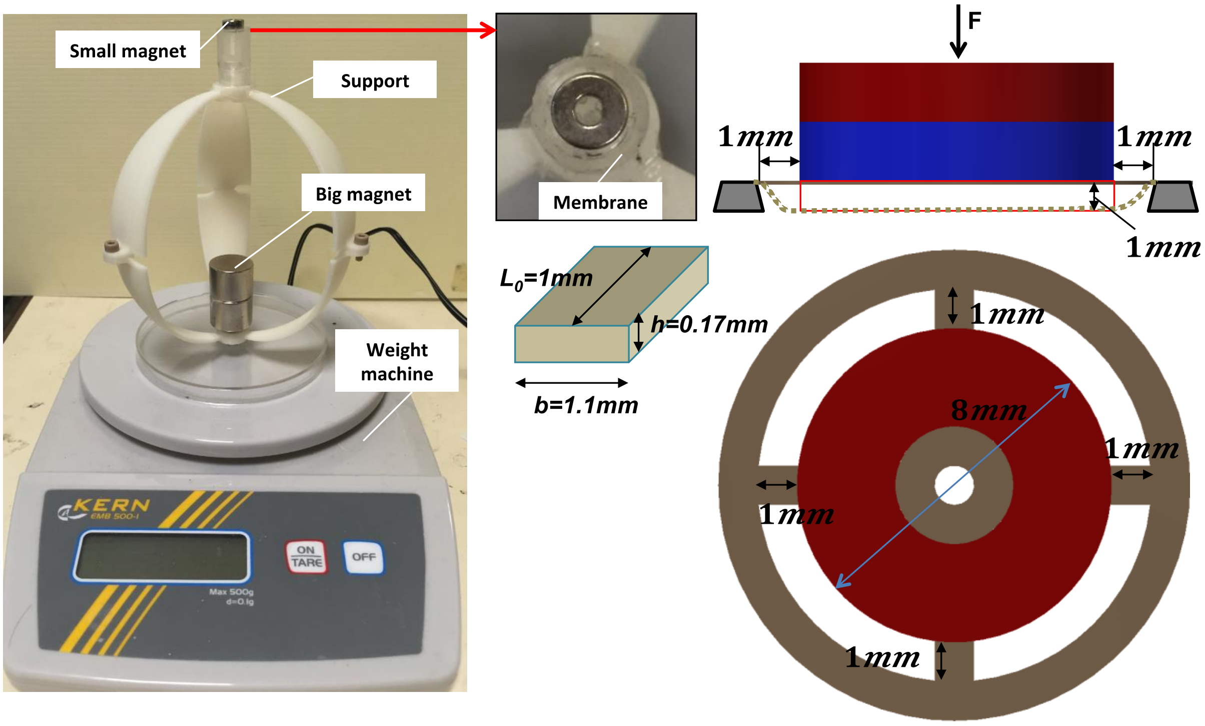

Fig. S2. Experiment to determine stiffness coefficient estimation K_θ for the flexible membrane

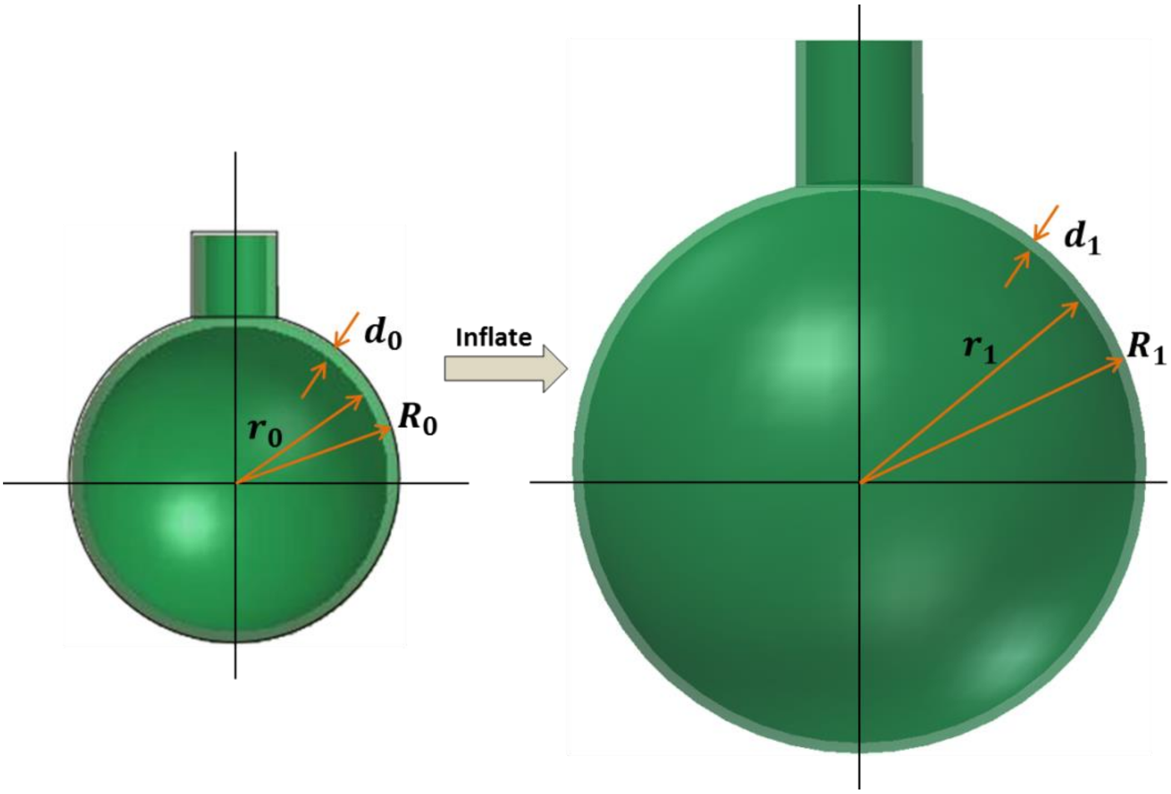

Fig. S3. Thickness estimation of the inflated Ecoflex balloon

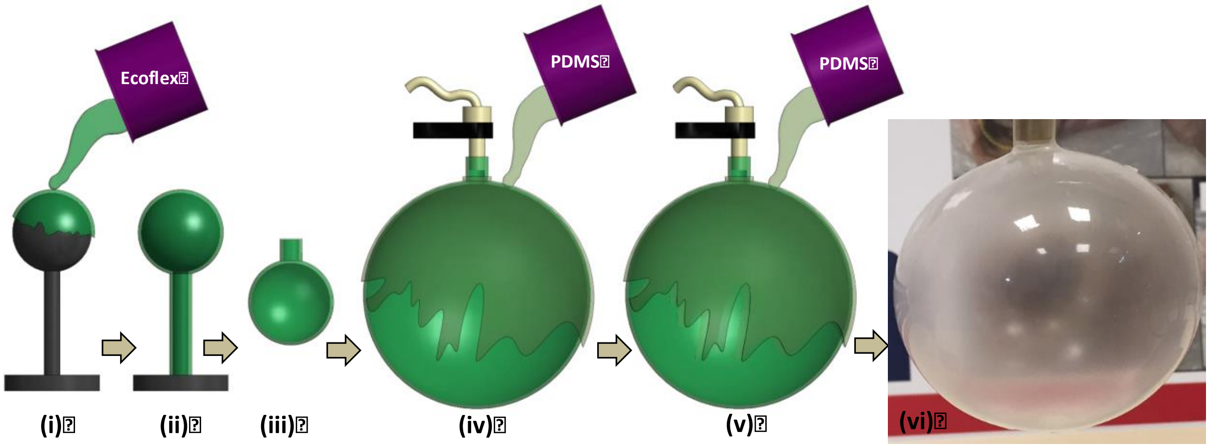

Fig. S4. Illustrated steps for making the 3D sphere PDMS balloon; (i) Pour the Ecoflex-0030 onto a spherical magnet of 25mm in diameter; (ii) Drain under the earth gravity and cure to form the sphere shell; (iii) Remove the Ecoflex shell from the sphere mold; (iv) Inflate the Ecoflex-0030 shell to a desired size and pour the liquid PDMS onto the inflated shell, subsequently cure the surface in the oven at 75°C for 4 hours ; (v) Invert inside-out the sphere balloon and pour PDMS onto the curve surface, subsequently cure the surface in the oven at 75°C for 4 hours; (vi) The obtained PDMS balloon.

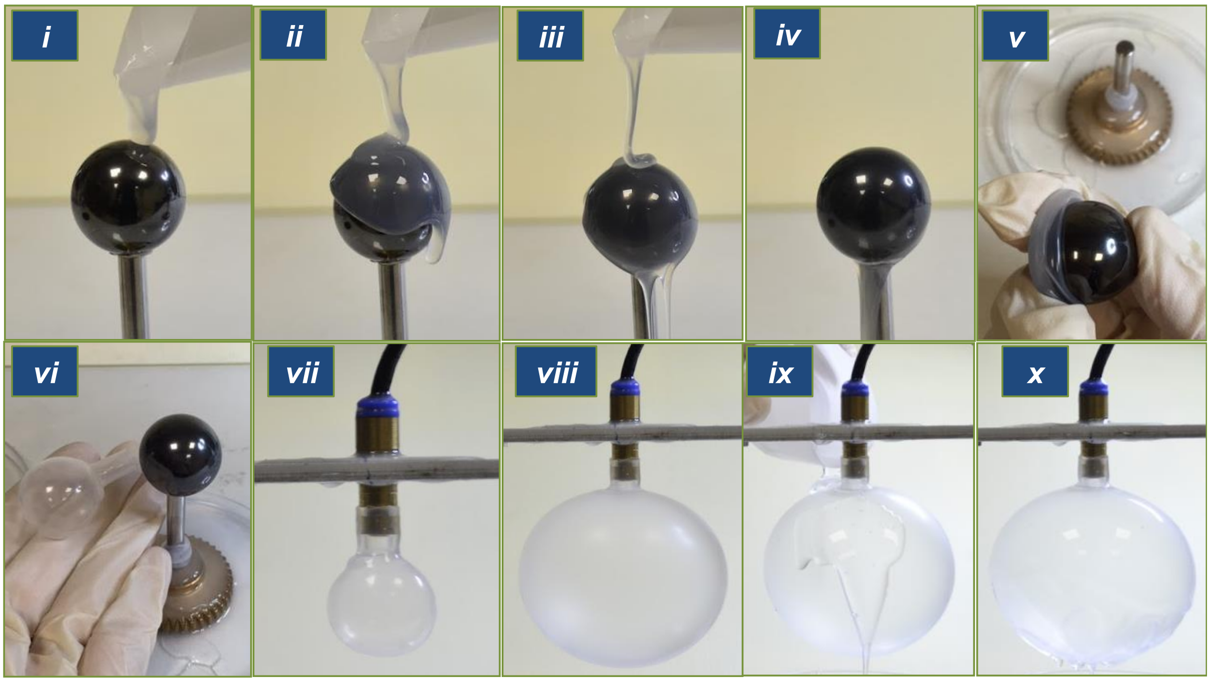

Fig.S5: Novel fabrication process for the spherical balloon with one outer layer of PDMS and one inner layer of Ecoflex with detailed process; (i-iv) Pour the liquid Ecoflex-0030 onto the sphere magnet; (v) peel off the elastic shell of the Ecoflex from the magnet; (vi) obtain the sphere Ecoflex balloon; (vii-viii) insert into the inflation system and inflate the balloon with air; (ix-x) pour liquid PDMS onto the inflated Ecoflex balloon and cure the PDMS layer.

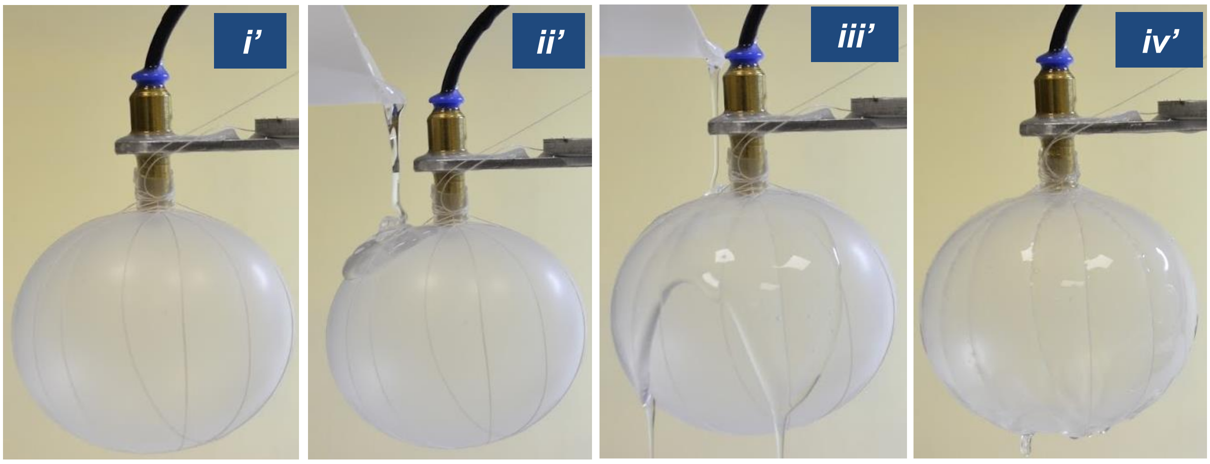

Fig. S6. Making a stronger IGB with one layer PDMS, one layer of Ecoflex, and an outer layer of sewing thread; (i') inflate the Ecoflex balloon and wrap the sewing thread; (ii'-iii') Pour liquid PDMS onto the inflated balloon; (iv') Drainage under the gravity to form the PDMS balloon

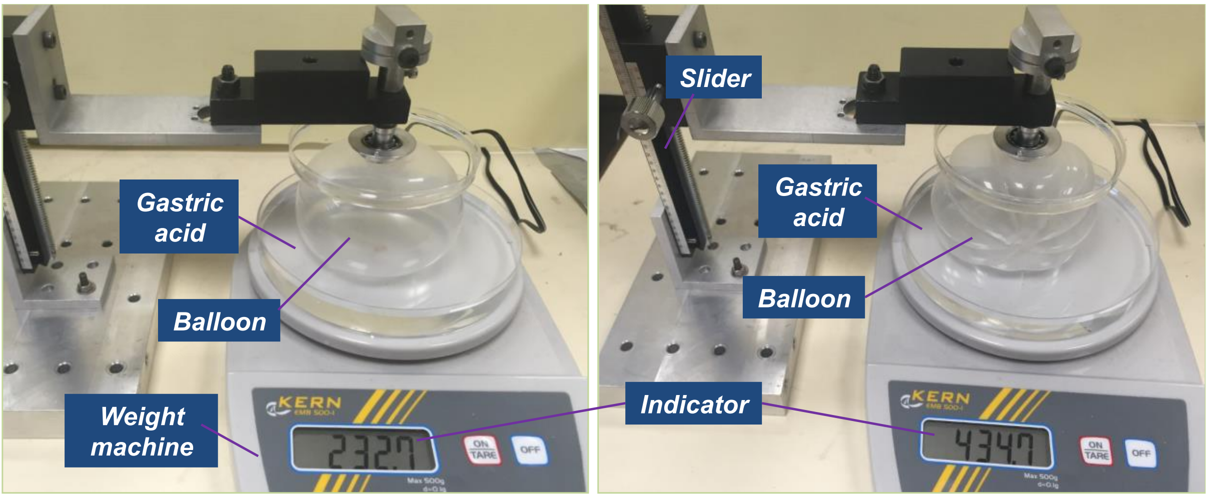

Fig. S7: Strength testing; (Left) with normal PDMS balloon; (Right) with sewing thread PDMS balloon

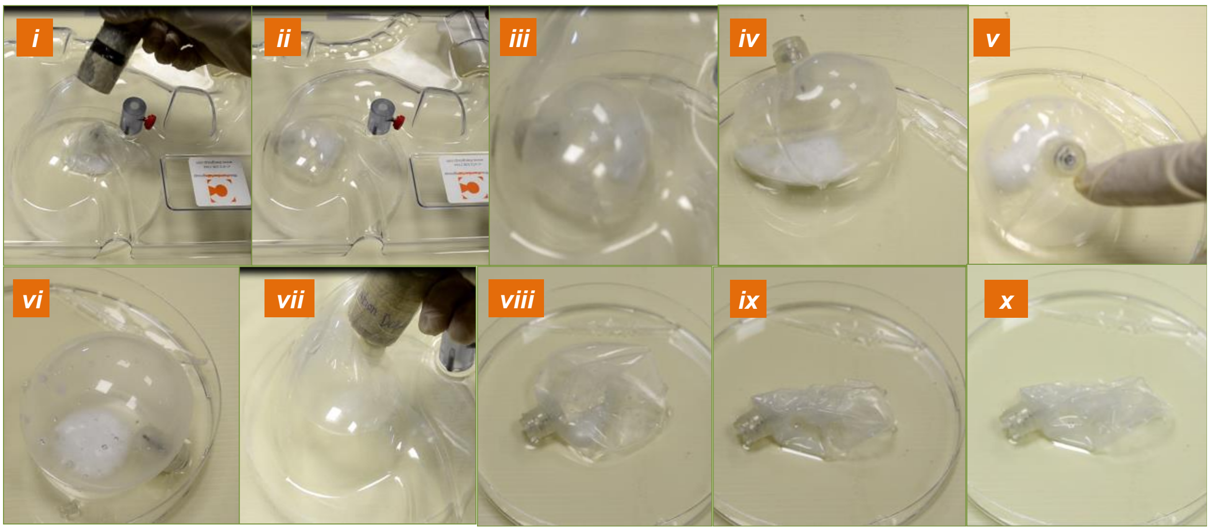

Fig. S8: Inflation and deflation for the balloon in simulated gastric acid environment; (i) Approach of the external magnet; (ii)-(iv) Inflation valve open, chemical reaction and CO_2 gas is created; (v) A few shakes are applied to stimulate the stomach motion; (vi) End of the reaction process; (vii) External magnet is introduced again; (viii)-(x) Deflation valve is opened and the balloon is completely deflated.

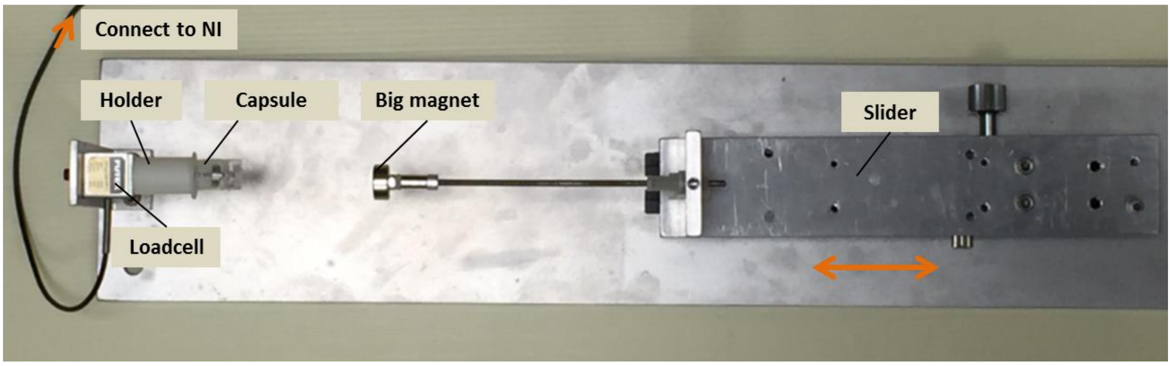

Fig. S9. Force measurement for the inflation and deflation with a FUTEK loadcell LSB200

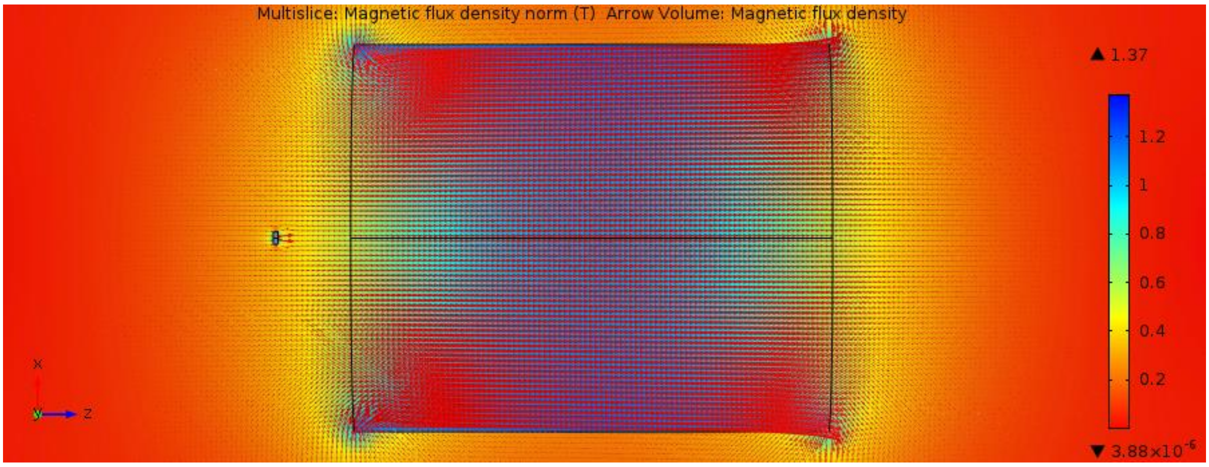

Fig. S10. COMSOL Multiphysics Simulation for Estimation of the External Magnet Size

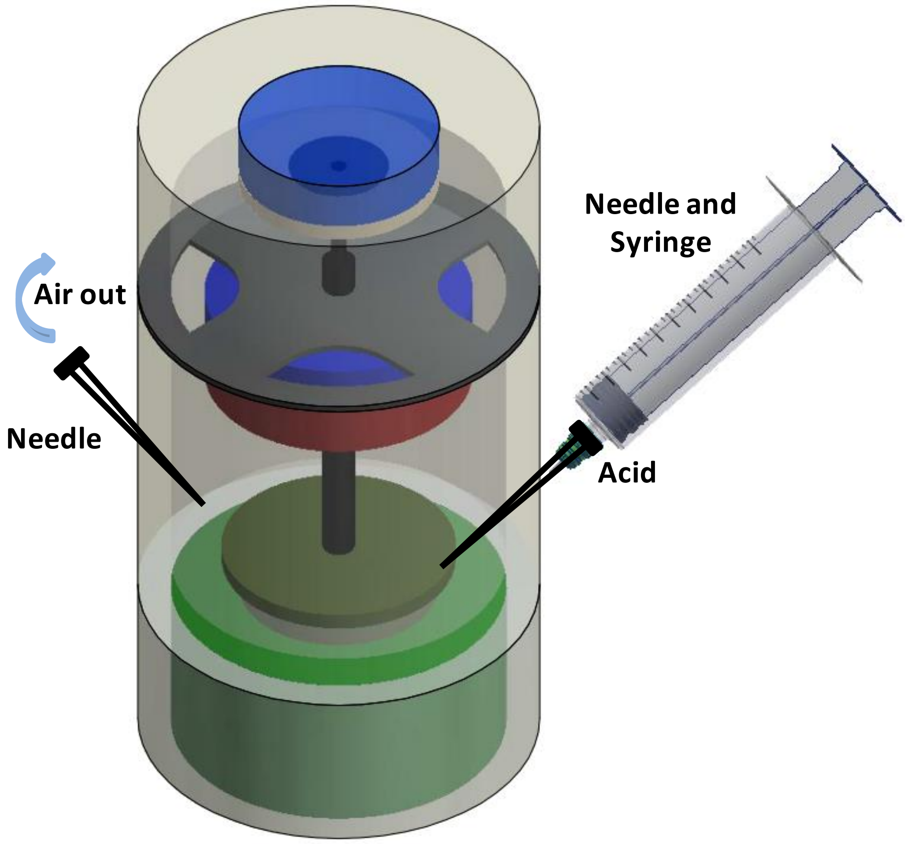

Fig. S11. Injection of the acid into the capsule; Step 1-Insert a needle into one side of the soft capsule; Step 2-Insert a needle-syringe with citric acid into the other side of the capsule; Step 3-Inject the acid into the capsule where the air inside will be released out of the capsule via the first needle.

Table S1: Force measurement for the inflation and deflation

Trials	Inflation force with membrane (N)	Deflation force with membrane (N)
1	0.563	0.470
2	0.573	0.485
3	0.561	0.466
4	0.558	0.467
5	0.577	0.478
Mean	0.5664	0.4732

Table S2: Soft capsule dimensions

Part’s names	Size
Capsule	Outer diameter:11mm; length: 24mm
PDMS membrane 6	Thickness h~0.1mm; width b~1.2mm
Inner ring magnet	Material: NdFeB/N52; Inner diameter:3mm; Outer diameter: 8mm, Thickness: 3mm
Fibre rod 5	Diameter: 0.254mm; Length 3mm; Material: carbon fibre
Fibre hollow rod 8	Inner diameter: 0.0279mm; Outer diameter: 0.7112mm; Length: 12mm ; Material: carbon fibre
Inflation valve 11	Diameter: 5mm; Thickness: 1.5mm; Material: PDMS
Deflation valve 4	Diameter: 2mm; Thickness: 1.5mm; Material: PDMS
Acid chamber	Inner diameter: 10mm; length: 15mm; Material: PDMS
Base chamber	Inner diameter: 10mm, length: 5mm; Material: PDMS
Outer balloon	Inflated diameter~70mm; Material: Ecoflex-0030 and PDMS

Table S3: Average Human Gastric Capacity

	Normal human	Obese human	After gastric band bass surgery	After using our capsule-balloon
Gastric capacity	~1200 ml [7-9]	1800 ml [7-9]	Reduce the stomach size of obese patients from 100ml to 600ml [10,11]	Depend on the patient's condition, our capsule-balloons is expected to occupy the stomach space from 150ml to 300ml
